# Systems of axon-like circuits for self-assembled and self-controlled growth of bioelectric networks

**DOI:** 10.1038/s41598-022-17103-4

**Published:** 2022-08-04

**Authors:** Russell Deaton, Max Garzon, Rojoba Yasmin

**Affiliations:** 1 Department of Electrical and Computer Engineering, University of Memphis, Memphis, TN 38152 USA; 2 Department of Computer Science, University of Memphis, Memphis, TN 38152 USA

**Keywords:** Organizing materials with DNA, Neural patterning, Developmental neurogenesis

## Abstract

By guiding cell and chemical migration and coupling with genetic mechanisms, bioelectric networks of potentials influence biological pattern formation and are known to have profound effects on growth processes. An abstract model that is amenable to exact analysis has been proposed in the circuit tile assembly model (cTAM) to understand self-assembled and self-controlled growth as an emergent phenomenon that is capable of complex behaviors, like self-replication. In the cTAM, a voltage source represents a finite supply of energy that drives growth until it is unable to overcome randomizing factors in the environment, represented by a threshold. Here, the cTAM is extended to the axon or alternating cTAM model (acTAM) to include a circuit similar to signal propagation in axons, exhibiting time-varying electric signals and a dependence on frequency of the input voltage. The acTAM produces systems of circuits whose electrical properties are coupled to their length as growth proceeds through self-assembly. The exact response is derived for increasingly complex circuit systems as the assembly proceeds. The model exhibits complicated behaviors that elucidate the interactive role of energy, environment, and noise with electric signals in axon-like circuits during biological growth of complex patterns and function.

## Introduction

Electric phenomena are essential in the development of complex biological structures and their function. By guiding cell migration^1^, electric fields and potentials influence wound healing and tissue regeneration^2^, and direct pattern formation (like left or right, up or down^3^) in processes such as early neuronal development^4^ or growth of plant roots^5^ and pollen tubes^6^. The aggregate effect of potential differences across membranes, gap junctions, action potentials in axons, and other bioelectric phenomena forms networks of electric potentials that communicate among cells to influence gene expression and thus, formation of biological structure and function in embryogenesis and morphogenesis^7–9^. Changes in physical structure are communicated electrically throughout the network, resulting in coordination of distant growth processes to produce spatially differentiated target structures^10^. The flow of information from the environment to biological structure and function through electric potentials represents computation without neurons and is postulated to be a primitive and ancient mechanism^11^.

Self-assembly models are inspired by autonomous interactions among component parts that build complex structures, including examples such as biomolecules (DNA and proteins), living organisms, social networks, and even galaxies. Theoretically, self-assembly is an algorithmic process, resulting in complex and powerful behavior that is capable of Turing universal computation^12,13^, and has produced new methods for building nanostructures^14^.

**Figure 1:**
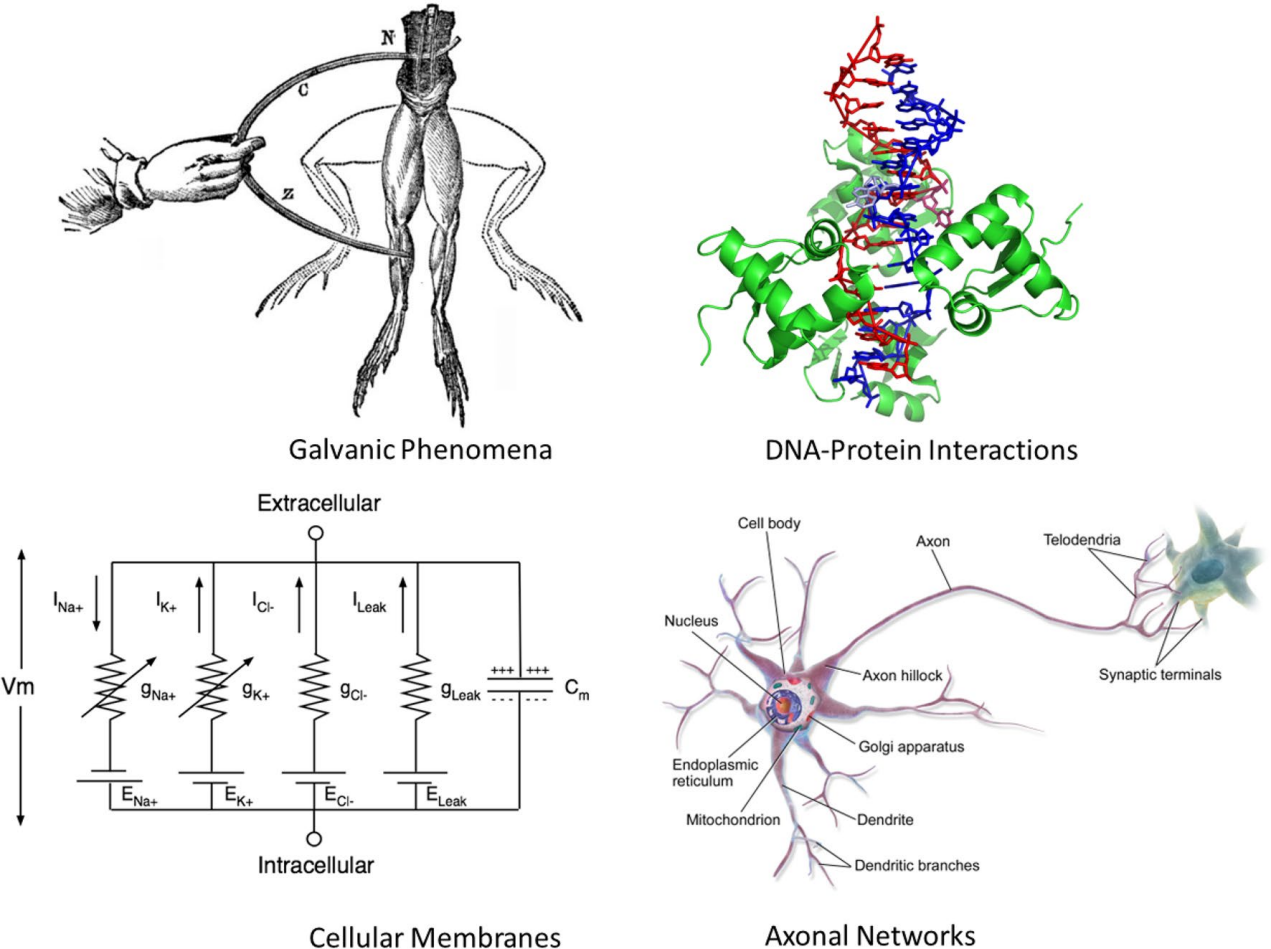
Examples of biological phenomena that could potentially be analyzed by the acTAM models and its variants to gain quantitative insights into their dynamical properties at the macrolevel (Galvanic Phenomena^19^, DNA–Protein Interactions^20^, Cellular Membranes^21^, Axonal Networks^22^) .

**Figure 2:**
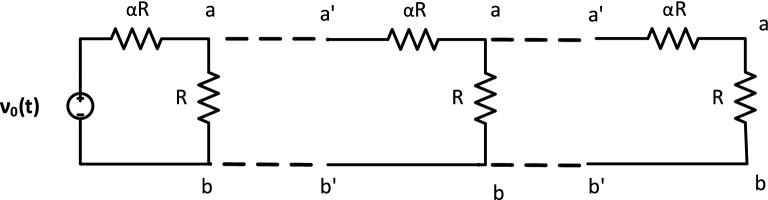
An cTAM ladder circuit is assembled as ladder tiles attach to a seed with a DC voltage source $\nu _0$ and subsequently, each other as long as the voltage across the resistor R at the terminus is at least $\tau$. In addition, Watson–Crick complementary DNA glues at the top (*a*–*a*$^{\prime }$) and bottom (*b*–*b*$^{\prime }$) bind the assembly together.

Originally motivated by DNA-based self-assembly^14^, the circuit tile assembly model (cTAM) was introduced to demonstrate how self-controlled growth^15^ and self-replication^16^ can be achieved as emergent properties without explicit programming. Rather, they are made possible by a finite resource (e.g., an electric potential $\nu _0$) that is consumed as tiles with simple circuits bind to a growing circuit assembly^15^, as illustrated in Fig. 2. In the cTAM, tiles attach if a constant voltage (Direct Current or DC) at the terminus is greater than or equal to a threshold $\tau$. This assembly process produces a family of circuits whose electrical properties change as the circuit grows, which in turn, modulates electric signals that are communicated throughout, as illustrated in Fig. 2. In contrast to models based on differential equations, the cTAM is abstract and discrete, with the potential to reveal the essence of electrical effects in biological phenomena. As an abstract model, the cTAM is amenable to analysis for exact prediction of its maximum circuit size through self-controlled growth and thus, of its electrical properties, as a function of the voltage source $\nu _0$ or threshold $\tau$^15,17,18^, and their effects on growth of the assembled circuit (such as self-replication^16^). Figure 1 shows examples of phenomena to which this model could be applied to gain quantitative insights into their dynamical properties at the macrolevel.

The goal of this paper is to further elucidate the role of electric signals in the growth of biological structures by adding a capacitor to the attaching tile in the cTAM model and extending the analysis to the frequency and time domains. The specific example biosystem that the cTAM represents can be regarded as an axon, and its electrically-driven growth as thresholded signal communication with its environment. Thus, this cTAM is termed the *axonal cTAM* (or just acTAM), which also denotes the time-varying nature of the model (‘ac’ for alternating current, or AC). The acTAM circuits approximate equivalent circuits for the propagation of action potentials in cable theory^23^ and the Hodgkin-Huxley model^24^. Propagation of electric signals along axons are the physical basis for communication from sensory to information-processing neurons and are fundamental to neural information processing. Moreover, axonal networks are capable of information processing^25,26^ and computation^27^ *without* neurons.

Capable of modeling both spiked and graded signals, acTAMs inherit a unique property of the cTAM models in that they produce a dynamically changing family of circuits in which both structure and electrical properties vary as growth proceeds. The acTAM captures the coupled effects of growth and electric potential in an axon-like circuit. In early neural circuit assembly, spontaneous electrical activity is important in development, which is followed by network refinement by signals evoked from sensory input^4,28^. Electrical activity also promotes neural repair^29^. Thus, the cTAM could also serve as a tool to understand the effect of electrical activity on the growth of axonal networks, as well as its role in the electric potential distributions that arise in bioelectric networks^8^ that lead to observable properties of form and function in biological organisms.

In what follows, the acTAM is defined, and the expressions for the node potentials as a function of the parameters and size of the circuit are derived Nodal analysis of acTAM systems and applied to the analysis of steady-state behavior. The transient and complete solutions are derived. The product of the acTAM is a system of ladder circuits, and their mature size is characterized. Finally, a discussion of the significance of the acTAM for development and function of structures in biological systems is presented.

## The axonal circuit tile assembly model (acTAM)

This section presents definitions of the cTAM (to make the paper self-contained) and its extension to the acTAM.

### Definition 1

*(acTAM Circuit)* An *axonal circuit* is a tuple

$$\begin{aligned} \Psi = (N, E, C, g, \partial N) \end{aligned}$$

on a graph (*N*, *E*), where *N* denotes the set of nodes corresponding to electrical nodes in the circuit, *E* denotes the set of edges, *C* is a set of circuit components (chosen from resistors, capacitors, and voltage sources) assigned to edges $e_{(i,j)} \in E$ where $\{i, j\} \in N$, and *g* maps some subset of nodes $\partial N$ to some subset of glues labeled from a finite alphabet $\Sigma$, i.e. $g: \partial N \rightarrow \Sigma$. $\partial N = N_{in} \cup N_{out}$ consists of two finite subsets of nodes, input nodes $N_{in}$ and output nodes $N_{out}$ of the circuit, and are the points at which glues bind tiles together on the boundary of the circuit. The size of the circuit is the number of tiles in it. (A cTAM is an acTAM with only resistor tiles, without capacitors.)

### Definition 2

*(acTAM)* An *axonal tile assembly system* (acTAM) is a tuple $\mathscr {C} = (\Gamma , S, \nu _0, \zeta , \tau )$, where $\Gamma$ is a finite nonempty alphabet of tiles, $S \subset \Gamma$ is a subset of seed tiles, $\nu _0$ is the potential at the power source, $\zeta : \Gamma (N_{in}) \times \Gamma (N_{out}) \rightarrow \{0,1\}$ is a glue indicator function that determines whether glues on input nodes of a tile match or bind to glues on output nodes of acTAM circuits, and $\tau \in {\mathbb {R}}_{+}$ is the threshold voltage that sets one of the criteria for further tile attachments.

**Figure 3:**
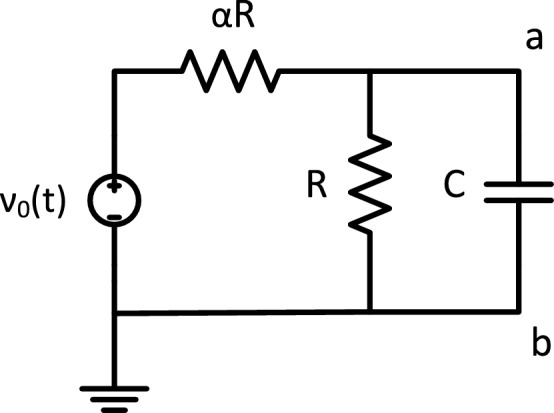
The seed tile for the acTAM consists of a voltage source $\nu _0(t)$, two resistors, *R* and $\alpha R$, and a capacitor *C*. The top node has glue *a*, and bottom node has glue *b*.

**Figure 4:**
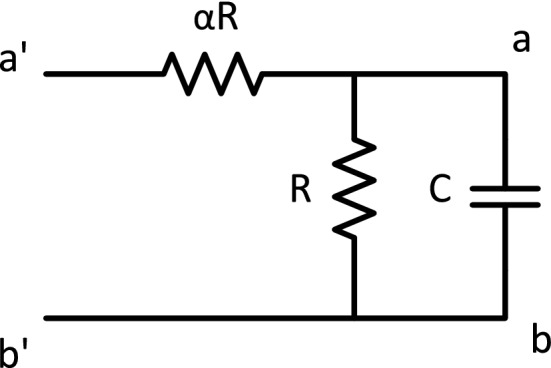
The ladder tile for the acTAM consists of resistors *R* and $\alpha R$, and a capacitor *C*. The input nodes have glues $a^{\prime }$ and $b^{\prime }$ on top and bottom and the output nodes have glues *a* and *b* on top and bottom, both respectively.

**Figure 5:**
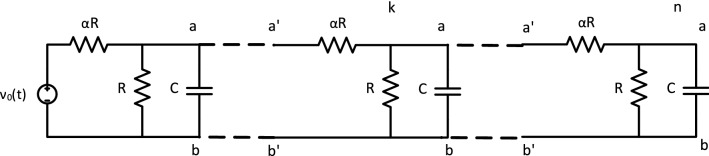
An acTAM ladder circuit is assembled as ladder tiles attach to a seed with a voltage source $\nu _0(t)$ and subsequently, each other as long as the voltage across the RC pair at the terminus is at least $\tau$. In addition, the hybridized glues at the top (a–a$^{\prime }$) and bottom (b–b$^{\prime }$) bind the assembly together. Nodes are labeled *k* and the terminal (last) node *n*.

The simplest acTAM consists of two circuit tiles, a seed tile (Fig. 3) and an unlimited number of copies of one ladder tile (Fig. 4). The circuit in the tiles consists of a resistor $\alpha R$ in series with a parallel combination of a capacitor *C* and a resistor *R*. The ladder circuit is equivalent to that used in cable theory^23^ to model signal propagation down axons, with $\alpha R$ being the longitudinal resistance, *R* the membrane resistance, and *C* the membrane capacitance. Growth of the ladder is determined by the electric potential difference across the RC pair, or between the nodes joining the $\alpha R$ resistors and the RC pair and the common ground (bottom of the circuit). Ladder tiles attach to the seed and subsequently, to other ladder tiles, if and only if the node potential at the output nodes of last tile in the assembly is at least $\tau$ (Fig. 5). The electric potential at node *k* in a given acTAM circuit of size *n* tiles at time *t* will be denoted $\nu ^n_k(t)$ (Or just $\nu _k(t)$ if a certain size *n* is assumed), where $k=0,\ldots ,n$, including the source potential $\nu _0^n(t)$ (or just $\nu _0$) at the seed.

In the simplest model, ladder tiles bind to form assemblies in the shape of growing ladders. In a *reaction-rate limited regime*, ladder tiles are present in saturation and always bind whenever the tip potential is at least $\tau$, whereas in a *diffusion-limited regime*, ladder tiles only arrive and attach at set time intervals. The specific mechanism by which tiles attach can be left unspecified under either assumption. One specific implementation would follow the well known aTAM model of DNA self-assembly^12,13^, in which a tile has a pair of oligonucleotides *a*, *b* on the output nodes of a DNA molecular tile that may bind to their corresponding Watson-Crick complements $a^{\prime }, b^{\prime }$ on input nodes of the attaching ladder tile. Other models can use protein-protein interactions resulting from electric potentials forming across ion channels^11^. In this paper, assembly processes are monotonic, i.e., once a tile is attached, the attachment will never dissolve. The behavior of the family of acTAM circuits is characterized below in general, for both DC and AC.

## Dynamics of network potentials in acTAM systems

**Figure 6:**
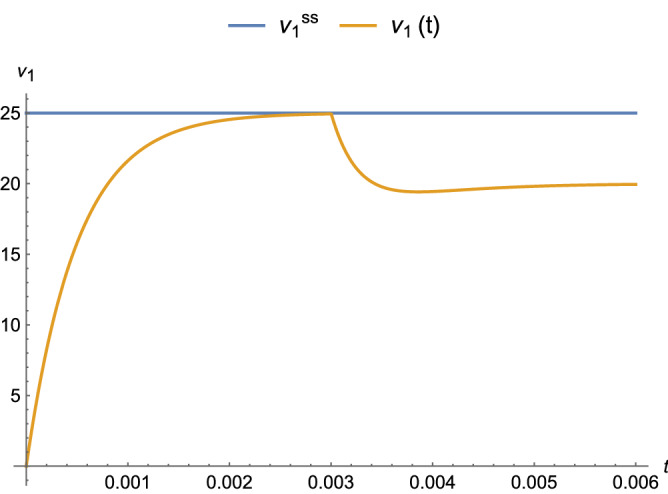
Response of first node potential in the seed tile for $\alpha = 1,\ R=1\ \Omega ,\ C=10^{-3}\ F$, and $|\nu _0| = 50\ \text {V}$. The second tile attaches to the seed at t = 0.003 s, causing the node potential at node 1 ($\nu _1$) to undergo a transient. The steady-state (ss) DC value 25 V (top line) for the seed alone is shown for reference.

Kirchoff’s Current Law (KCL) is about conservation of charge and states that the sum of the currents entering and exiting any node in a circuit must be 0. In particular, the seed (Fig. 3) has a distribution of potentials at its three nodes (source $\nu _0$, ground $\nu _{-1}$, and node potential $\nu _1$ at the tip of the tile (between $\alpha R$ and the parallel *C*–*R* pair). Attachment of successive ladder tiles causes a (speed of light, nearly instantaneous for relatively small circuits) propagation of the signals to the other tiles, which reconfigures the node potentials at the previous nodes into a new steady state after a brief transient, as illustrated in Fig. 6. Over time, the self-assembly process in an acTAM model generates a family of circuits of increasing size (number of tiles) with a dynamic potential distribution $\nu ^n_k$ at nodes *k* in a ladder of size *n* ($k\in \{1,\ldots ,n\}$). Because of the series-parallel resistance of the circuits in the family, the tip potentials $\nu ^n_n$ are a decreasing function of time or size, so they eventually become unable to support new attachments, as shown in the DC case in^15,17^. Thus an acTAM really defines a dynamical system of growing circuits of increasing size that exhibit emergent characteristic behavior.

**Figure 7:**
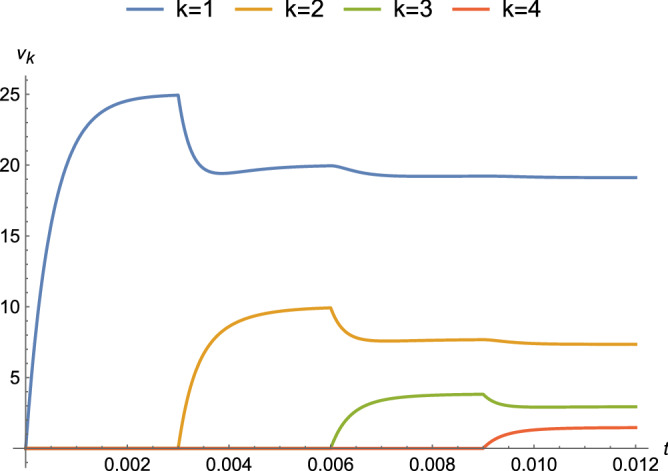
Response of node potentials for parameters $\alpha = 1,\ R=1\ \Omega ,\ C = 10^{-3}\ F$, and $|\nu _0| = 50\ \text {V}$. Tiles attach every t = 0.003 s, causing the node potentials to undergo transients before returning toward steady-state values. With a threshold of $\tau = 3\ \text {V}$, no further attachments occur past node $k=4$.

For the analysis of the acTAM, the differential equations for a system of circuits are derived using nodal analysis (KCL). First, the system is solved for a non-time-varying (DC) case, which makes the capacitor an open circuit and the circuit purely resistive (“Nodal analysis of acTAM systems”). Then, in “Steady-state phasor analysis”, the steady-state response in the frequency domain is derived from the DC equations with complex impedances. Thus, the steady-state time response is derived for a sinusoidal input voltage. The transient response (“Transient response”) was determined from results in^30^ and initial conditions, which are set by the node potentials when a new ladder tile attaches. The complete response is steady-state plus transient (“Complete solution”). The dynamic behavior is shown in Fig. 7.

### Nodal analysis of acTAM systems

The temporal dynamics of the circuit system assembled in the acTAM model can be characterized in general using the equations for the node voltages $\nu ^n_k$ obtained from Kirchoff’s Current Law (KCL). (We will drop the superindex *n* when the context is not ambiguous.) Given the circuit in Fig. 5, for an arbitrary intermediate node *k*, where the current in the capacitor is $C d \nu _k / d t$, the node equation is

1 $$\begin{aligned} (2 + \alpha ) \nu _k - \nu _{k-1} - \nu _{k+1} + \alpha R C \frac{d \nu _k}{d t} = 0, \end{aligned}$$

where $\nu _k$ is the electric potential at node *k*. Rearranging and using the shorthand $\nu _k^{\prime } = d \nu _k/d t$, the differential equation for $\nu _k$ is

2 $$\begin{aligned} \nu _k^{\prime } = \frac{\nu _{k-1}}{\alpha R C} - \frac{2+\alpha }{\alpha R C} \nu _k + \frac{\nu _{k+1}}{\alpha R C}. \end{aligned}$$

For the first node $k=1$, the equation is

3 $$\begin{aligned} \nu _1^{\prime } = \frac{\nu _{0}(t)}{\alpha R C} - \frac{2+\alpha }{\alpha R C} \nu _1 + \frac{\nu _{2}}{\alpha R C}, \end{aligned}$$

and for the last node $k=n,$

4 $$\begin{aligned} \nu _n^{\prime } = \frac{\nu _{n-1}(t)}{\alpha R C} - \frac{1+\alpha }{\alpha R C} \nu _n. \end{aligned}$$

In matrix form, the system of differential equations thus becomes

5 $$\begin{aligned} \begin{bmatrix} \nu _1^{\prime }\\ \vdots \\ \nu _{k}^{\prime }\\ \vdots \\ \nu _n^{\prime } \end{bmatrix} = \begin{bmatrix} -\frac{2+\alpha }{\alpha R C}&{}\frac{1}{\alpha R C}&{}0&{}\cdots &{}0\\ \frac{1}{\alpha R C}&{}\ddots &{}\ddots &{}\ddots &{}\vdots \\ \vdots &{}\frac{1}{\alpha R C}&{}-\frac{2+\alpha }{\alpha R C}&{}\frac{1}{\alpha R C}&{}\vdots \\ \vdots &{}\ddots &{}\ddots &{}\ddots &{}\vdots \\ 0&{}\cdots &{}\cdots &{}\frac{1}{\alpha R C}&{}-\frac{1+\alpha }{\alpha R C} \end{bmatrix} \begin{bmatrix} \nu _1\\ \vdots \\ \nu _{k}\\ \vdots \\ \nu _n \end{bmatrix} + \begin{bmatrix} \nu _0(t)\\ 0\\ \vdots \\ \vdots \\ \vdots \\ \end{bmatrix}. \end{aligned}$$

Under direct current (DC, time invariant supply), the capacitor C is an open circuit and can be ignored, and thus, the general form of the node equations for node *k* of *n* is

6 $$\begin{aligned} (2 + \alpha ) \nu _k - \nu _{k-1} - \nu _{k+1} = 0. \end{aligned}$$

The set of equations then becomes

7 $$\begin{aligned} {\mathbf {A}}_n \mathbf {\nu } = {\mathbf {b}}_n \,, \end{aligned}$$

where the matrix of coefficients is

8 $$\begin{aligned} {\mathbf {A}}_n = \begin{bmatrix} 2+\alpha &{}-1&{}0&{}\cdots &{}0\\ -1&{}\ddots &{}\ddots &{}\ddots &{}\vdots \\ \vdots &{}-1&{}2+\alpha &{}-1&{}\vdots \\ \vdots &{}\ddots &{}\ddots &{}\ddots &{}\vdots \\ 0&{}\cdots &{}\cdots &{}-1&{}1+\alpha \end{bmatrix}, \end{aligned}$$

and the source vector is

9 $$\begin{aligned} {\mathbf {b}}_n = \begin{bmatrix} \nu _0\\ 0\\ \vdots \end{bmatrix}, \end{aligned}$$

with $\nu _0$ the DC source potential. According to Cramer’s rule, the node potentials are then given by

10 $$\begin{aligned} \nu _k = \frac{|{\mathbf {A}}^{(k)}_n|}{|{\mathbf {A}}_n|} = \frac{A^{(k)}_n}{A_n}, \end{aligned}$$

where ${\mathbf {A}}^{(k)}_n$ is the matrix with the *k*-th column of ${\mathbf {A}}_n$ (Eq. 8) replaced with the vector ${\mathbf {b}}_n$ from Eq. (9), and $|\cdot |$ or a corresponding capital letter denotes the determinant of the corresponding matrix. Since ${\mathbf {A}}_n$ is a tridiagonal matrix for $n \ge 2$, the determinants $A_n = |{\mathbf {A}}_n|$ satisfy a recurrence relation

11 $$\begin{aligned} A_{k} = (2+\alpha ) A_{k-1} - A_{k-2} \end{aligned}$$

with $A_1 = 1+\alpha$, and $A_{0} = 0$. Equation (11) is a linear recurrence, and has a solution similar to that derived in^31^ for equivalent resistance. Therefore, the general solution for a circuit ladder of size *n* is a linear combination

12 $$\begin{aligned} A_n = a_1 \rho _1^n + a_2 \rho _2^n, \end{aligned}$$

where $a_1$ and $a_2$ are constants, and $\rho _1$ and $\rho _2$ are the roots of the characteristic equation of the recursion,

13 $$\begin{aligned} \rho ^2 - (2+\alpha ) \rho + 1 = 0. \end{aligned}$$

Solving Eq. (13) yields two solutions,

14 $$\begin{aligned} \rho _1&= \frac{(2+\alpha ) + \sqrt{\alpha (\alpha + 4)}}{2} \nonumber \\ \rho _2&= \frac{(2+\alpha ) - \sqrt{\alpha (\alpha + 4)}}{2}. \end{aligned}$$

Using the the initial conditions from Eq. (11) with $k=1$ and $k=2$,

$$\begin{aligned} A_1&= 1 + \alpha \\ A_2&= 1 + 3 \alpha + \alpha ^2 \end{aligned}$$

yields the following two equations,

$$\begin{aligned}&a_1 \rho _1 + a_2 \rho _2 = 1+\alpha \\&a_1 \rho _1^2 + a_2 \rho _2^2 = 1 + 3 \alpha + \alpha ^2. \end{aligned}$$

Solving for $a_1$ and $a_2$ gives

15 $$\begin{aligned} a_1&= \frac{4+\alpha +\sqrt{\alpha (4+\alpha )}}{2 (4+\alpha )} \nonumber \\ a_2&= \frac{1}{2}-\frac{\alpha }{2\sqrt{\alpha (4+\alpha )}}. \end{aligned}$$

The recurrence for $$A_n^{(k)}=|{\mathbf 
{A}}_{n}^{k}|$$ is

16 $$\begin{aligned} A_n^{(k)} = \nu _0 A_{n-k}, \end{aligned}$$

where

17 $$\begin{aligned} A_n^{(k)} = \nu _0 (a_1 \rho _1^{n-k} + a_2 \rho _2^{n-k}), \end{aligned}$$

with $a_1$ and $a_2$ given by Eq. (15). Therefore, according to Cramer’s rule (Eq. 10), the node potentials at node *k* are

18 $$\begin{aligned} \nu _k = \nu _0 \frac{A_n^{(k)}}{A_n}, \end{aligned}$$

and for the last (terminal) node ($k=n$) in the ladder, the node potential is

19 $$\begin{aligned} \nu _n = \frac{\nu _0}{A_n}, \end{aligned}$$

where $\nu _0$ is the source voltage.

Since $|\rho _2| < 1$, as *n* becomes large, the node potential at the terminus of the ladder in the last tile is bounded by

20 $$\begin{aligned} \nu _n < \nu _0 \frac{1}{a_1 \rho _1^n} = \nu _0 \frac{\left( 2^{n+1}(4+\alpha )\right) }{\left( 2+\alpha +\sqrt{\alpha (4+\alpha )}\right) ^n \left( 4+\alpha +\sqrt{\alpha (4+\alpha )}\right) }. \end{aligned}$$

### Steady-state phasor analysis

With a sinusoidal time-varying source $\nu _0(t)$, phasor analysis can be used to derive the steady state response of the circuit in Fig. 5. Phasor analysis represents circuit components with complex impedances, allowing the steady state behavior of the circuit to be solved using nodal analysis, as in the DC case. The impedances are $Z_1 = R/(1 + j \omega R C)$ for the parallel combination of *R* and *C* and $Z_2 = \alpha R$ for the series resistor. For an intermediate node *k* other than 1 or *n*, the node equation is

21 $$\begin{aligned} \frac{\nu _k-\nu _{k-1}}{Z_2} + \frac{\nu _k}{Z_1} + \frac{\nu _k-\nu _{k+1}}{Z_2} = -\nu _{k-1} + (2 + \alpha (1 + j \omega R C)) \nu _k - \nu _{k+1} = 0, \end{aligned}$$

with $\omega$ the angular frequency. If we let $\alpha ^{\prime } = \alpha (1 + j \omega R C)$, then, the system of equations from nodal analysis is identical to those for DC (Eq. 7), except the node potentials are now in the phasor or frequency domain. Therefore, with that substitution, the solution for node potentials is the same as Eq. (17),

22 $$\begin{aligned} \nu _k = \nu _0 \frac{A_n^{(k)}}{A_n}, \end{aligned}$$

with complex

23 $$\begin{aligned} \rho _1 =&\frac{1}{2} \left( 2+\alpha +j \omega \alpha R C+\sqrt{(\alpha +j \omega \alpha R C) (4+\alpha +j \omega \alpha R C)}\right) \nonumber \\ \rho _2 =&\frac{1}{2} \left( 2+\alpha +j \omega \alpha R C-\sqrt{(\alpha +j \omega \alpha R C) (4+\alpha +j \omega \alpha R C)}\right) , \end{aligned}$$

and

24 $$\begin{aligned} & a_{1} = \frac{{4 + \alpha + j\omega \alpha RC + \sqrt {(\alpha + j\omega \alpha RC)(4 + \alpha + j\omega \alpha RC)} }}{{2(4 + \alpha + j\omega \alpha RC)}} \\ & a_{2} = \frac{1}{2} - \frac{{\alpha + j\omega \alpha RC}}{{2\sqrt {(\alpha + j\omega \alpha RC)(4 + \alpha + j\omega \alpha RC)} }}. \\ \end{aligned}$$

in which $\alpha ^{\prime }$ has been substituted for $\alpha$. The node potential at the terminus of the ladder is bounded by Eq. (20) with $\alpha ^{\prime }$ substituted for $\alpha$,

25 $$\begin{aligned} \nu _n < \nu _0(\omega ) \frac{\left( 2^{n+1}(4+\alpha ^{\prime })\right) }{\left( 2+\alpha ^{\prime }+\sqrt{\alpha ^{\prime } (4+\alpha ^{\prime })}\right) ^n \left( 4+\alpha ^{\prime }+\sqrt{\alpha ^{\prime } (4+\alpha ^{\prime })}\right) }. \end{aligned}$$

where the source voltage is now a function of frequency $\omega$. Converting from frequency back to the time domain, the steady state node voltages are

26 $$\begin{aligned} \nu ^{\text {ss}}_{k}(t) = |\nu _k| \cos (\omega t + \angle \nu _k(\omega )), \end{aligned}$$

where $|\nu _k|$ is the magnitude and $\angle \nu _k$ is the phase of the complex node potentials $\nu _k(\omega )$. Like axons, the acTAM ladder is a low-pass filter. The magnitude of the frequency response (Eq. 22) and phase are shown in Figs. 8 and 9, respectively.

**Figure 8:**
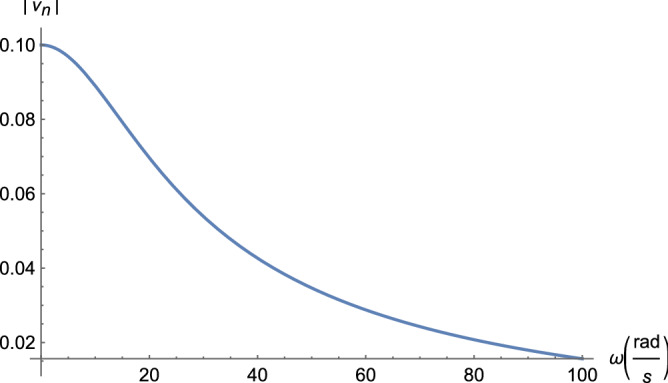
Magnitude of the frequency response for parameters that approximate an axon ($\alpha = 10^{-6},\ R = 10^{12}\ \Omega ,\ C = 10^{-8}\ F$, and $|\nu _0| = 100\ \text {mV}$).

**Figure 9:**
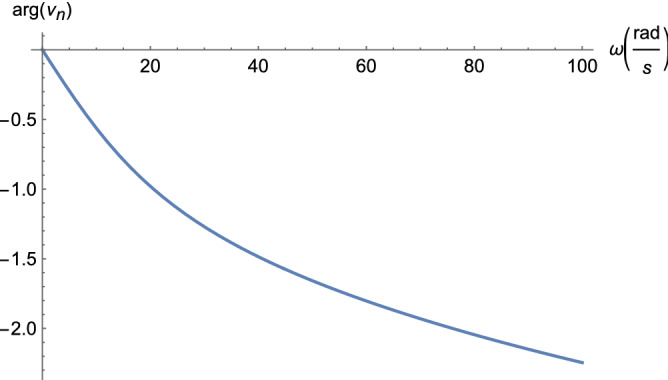
Phase of the frequency response for parameters that approximate an axon ($\alpha = 10^{-6},\ R = 10^{12}\ \Omega ,\ C = 10^{-8}\ F$, and $|\nu _0| = 100\ \text {mV}$).

### Transient response

According to^30^, the eigenvalue for node *k* of *n* for the matrix in Eq. (8) with $\alpha ^{\prime }$ substituted for $\alpha$ is

27 $$\begin{aligned} \lambda _k = \frac{-(2+\alpha +j \omega \alpha R C)}{R C (\alpha +j \omega \alpha R C)}+\frac{2 \cos \left( \frac{(2 k - 1) \pi }{2 n + 1}\right) }{R C (\alpha +j \omega \alpha R C)}, \end{aligned}$$

and the *i*th value in the *k*th eigenvector $\chi _{i,k}$ for $\lambda _k$ is

28 $$\begin{aligned} \chi _{i,k} = \sin \left( \frac{i (2 k - 1) \pi }{2 n + 1} \right) . \end{aligned}$$

The transient response for node *k* of *n* is thus

29 $$\begin{aligned} \nu ^{\text {tr}}_k(t) = \sum _{i=0}^{n} b_i \chi _{i,k} \exp (\lambda _i t), \end{aligned}$$

where the $b_i$’s are determined from initial conditions.

### Complete solution

With a sinusoidal time-varying source $\nu _0(t)$, the complete solution for a node $k \in \{1,\ldots ,n\}$ is the sum of the transient (Eq. 29) and steady-state (Eq. 26) responses,

30 $$\begin{aligned} \nu ^n_k(t) = \nu ^{\text {tr}}_k(t)+ \nu ^{\text {ss}}_k(t), \end{aligned}$$

for a ladder of length *n*. The constants $b_i$ in Eq. (29) are determined by initial conditions, which are the node potentials when a new tile attaches or are the values of the potentials $\nu ^{n-1}_k(t)$ for nodes $k \in \{1,\ldots ,n-1\}$ in a ladder of length $n-1$, i.e. the node potentials ($\nu ^{n-1}_k(t)$) in the ladder of length $n-1$ are the initial conditions for the node potentials ($\nu ^n_k(t)$) in a ladder of length *n*. For an attaching tile, since it is disconnected from a source, the initial condition is $\nu ^{n}_n(0)=0$. If attachments to a growing ladder only occur after it has practically reached steady state, then, the initial conditions for attachment to a ladder of size *n* are the DC values for the node potentials, or $\nu ^n_k$ (Eq. 18).

**Figure 10:**
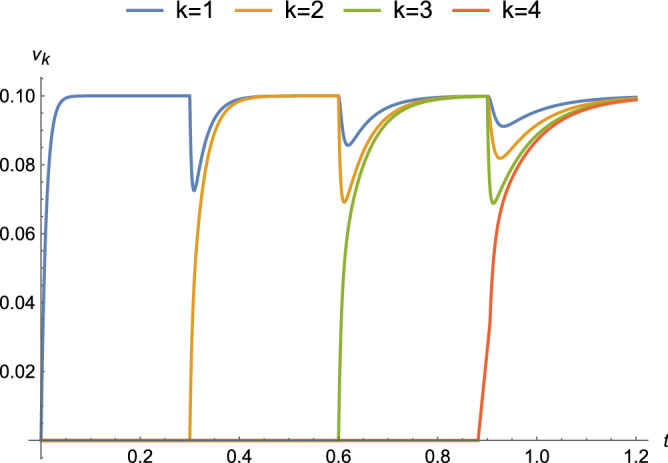
Complete response for parameters that approximate an axon ($\alpha = 10^{-6},\ R=10^{12}\ \Omega ,\ C=10^{-8}\ F$, and $|\nu _0| = 100\ \text {mV}$) under a binding regime that approximates diffusion-limited growth with a time between attachments of $T = 0.3$ s.

**Figure 11:**
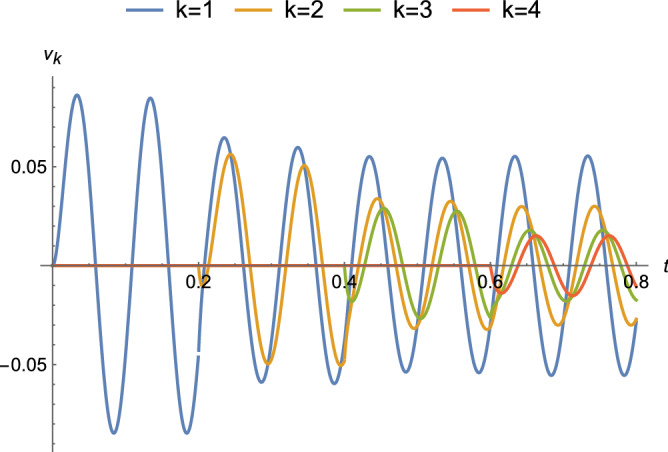
Complete response for parameters that approximate an axon ($\alpha = 10^{-6},\ R = 10^{12}\ \Omega ,\ C=10^{-8}\ F$, and $\nu _0(t) = 100 \sin (\omega t + \phi )\ \text {mV}$ with $\omega = 20 \pi$ (rad/s)) under binding regime that approximates diffusion-limited growth with a time between attachments of $T = 0.2$ s.

**Figure 12:**
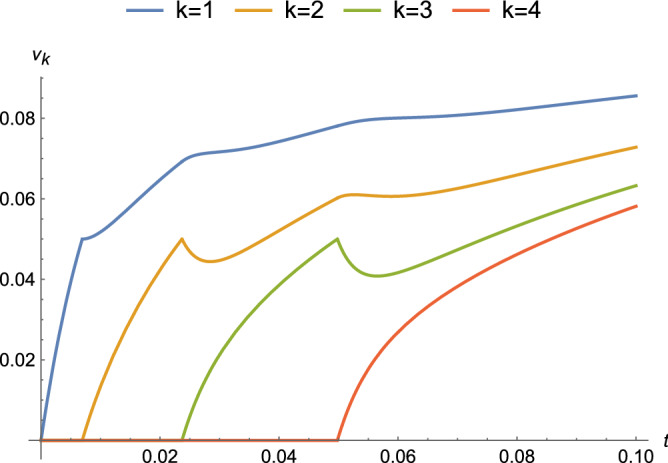
Complete response for parameters that approximate an axon ($\alpha = 10^{-6},\ R=10^{12}\ \Omega ,\ C=10^{-8}\ F$, and $\nu _0(t) = 100\ \text {mV}$) under binding regime that approximates reaction-rate limited growth with tiles in saturation and a threshold of $\tau = 50\ \text {mV}$.

Equation (30) for different nodes *k* is plotted for a couple of binding regimes. The first binding regime is an approximation for diffusion-limited growth in which tiles are available for binding at a fixed time interval *T*. Every *T* seconds, a new tile arrives at a growing ladder and attaches if the terminal potential is at least $\tau$. In Fig. 10, with circuit parameters that approximate those for axons, a DC voltage source is applied at $t=0$ and tiles arrive every $T = 0.3$ s. There is relatively little attenuation as growth proceeds because the value of $\alpha$ is small compared to *R*. The same circuit parameters and $T = 0.2$ s are used in Fig. 11 with $\nu _0(t) = 0.1 \sin (\omega t + \phi )$ and $\omega = 20 \pi$ (rad/s). An exponential decrease in amplitude is observed, as well as shifts in the phase of the sinusoid. The other regime approximates reaction-rate limited growth in which tiles are available in saturation and is shown in Fig. 12. As soon as the terminal potential $\nu _n \ge \tau$, a new tile will attach. Because the time to reach $\tau$ is shorter than the time to reach steady-state, the response at each node is attenuated to a transient value at the attachment and changes to new time behavior with $\nu ^{n-1}_{k}(t)$ as the initial condition, where for the last tile $\nu ^{n}_n(0) = 0$ and for the next to last tile $\nu ^{n}_{n-1}(0)=\tau$.

## Bounds

Using the expressions derived in previous sections, bounds on the length of the ladder can be derived. Given the biological connection of the acTAM ladder circuit and growth/signal propagation in axons, the length provides a measure of both the driving potential $\nu _0$ and the threshold $\tau$. In our model, $\nu _0$ represents the source of energy for the growth or the strength of the signal from a sensor, and the threshold $\tau$ represents those randomizing forces in the environment that oppose either growth or signal propagation. Thus, the length of the ladder is a measure of both.

To determine bounds on the length *n* of the ladder, the terminal voltage has to be less than the threshold, i.e.,

31 $$\begin{aligned} \nu _n = \nu _0 \frac{1}{A_n} < \tau , \end{aligned}$$

where the expression for $\nu _n$ is from Eq. (19). Rearranging, it is found that $A_n > \nu _0/\tau$, which has no ready solution for *n*. Nevertheless, by using Eq. (20) for *n* large, it is found that the condition for growth to cease is bounded by

32 $$\begin{aligned} n > \frac{\log {\frac{\nu _0}{2 \tau } (4+\alpha ^{\prime } -\sqrt{\alpha ^{\prime } (4+\alpha ^{\prime })})}}{\log {\rho _1}}. \end{aligned}$$

**Figure 13:**
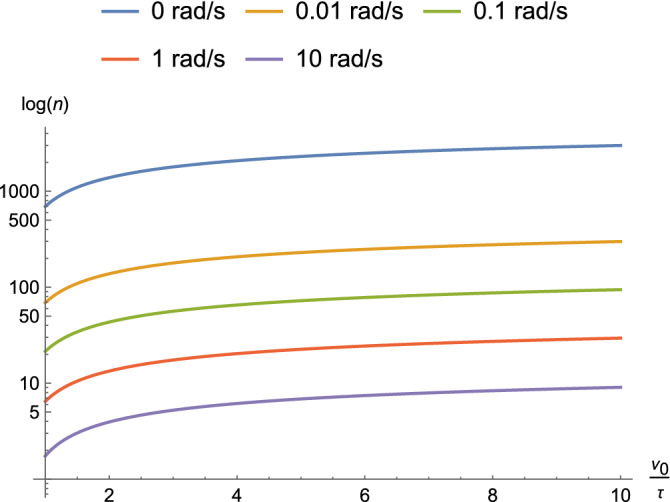
Dependence of length *n* on $\nu _0/\tau$ at different frequencies for parameters that approximate an axon ($\alpha = 10^{-6},\ R = 10^{12}\ \Omega ,\ C = 10^{-8}\ F$, and $\nu _0(t) = 100\ \text {mV}$).

Thus, the growth is bounded and the length given $\nu _0$ and $\tau$ can be estimated. Moreover, for complex $\alpha ^{\prime }$, length is not only modulated by input potential and threshold, but also by the frequency of the input. The dependence of length *n* on frequency is shown in Fig. 13.

Likewise, the input potential $\nu _0$ and threshold $\tau$ that generate a given length can be determined from Eq. (31) as

33 $$\begin{aligned} \frac{\nu _o}{\tau } < A_n. \end{aligned}$$

For $\alpha ^{\prime } = 1$ in Eq. (18), $A_n$ becomes

34 $$\begin{aligned} \frac{(-5+\sqrt{5})(3+\sqrt{5})^n+2(3-\sqrt{5})^n(-5+2\sqrt{5})}{2^n (5 (-3+\sqrt{5}))}. \end{aligned}$$

By using relations for the golden ratio $\phi$ in Eq. (34), Eq. (33) becomes

35 $$\begin{aligned} \frac{\nu _o}{\tau } < F_{2n+1}, \end{aligned}$$

where $F_{2n+1}$ are the odd Fibonacci numbers, which is similar to a result for the equivalent resistance of long resistive ladders^32^. The golden ratio and Fibonacci numbers appear frequently in nature, mathematics, and human designs. Thus, the relationship of driving force $\nu _0$ and environment $\tau$ that produce a given length ladder to the golden ratio and the Fibonacci sequence provide further evidence of the connection of the acTAM to biological systems.

In addition, using the equation for the node potential (Eq. 18) and recurrence for the determinant (Eq. 11),

36 $$\begin{aligned} \frac{\nu _0}{\tau } < (2+\alpha ^{\prime }) A_{n-1} - A_{n-2}, \end{aligned}$$

it is seen that this ratio is bound by a Lucas sequence *U*(*P*, *Q*) with $P=2+\alpha ^{\prime }$, $Q=1$, $U_1(P,Q) = 1$, and $U_0(P,Q) = 0$. Comparing $\nu _0/\tau$ to generate lengths *n* and $n-1$ as *n* becomes large gives

37 $$\begin{aligned} A_n/A_{n-1} = \frac{1}{2} (2 + \alpha ^{\prime } + \sqrt{\alpha ^{\prime } (4+\alpha ^{\prime })}), \end{aligned}$$

which is one of the solutions, $\rho _1$, to the characteristic (Eq. 14), the limit of the ratio of two numbers in the Lucas sequence, and for $\alpha ^{\prime } = 1$ is equal to $1 + \phi$, where $\phi$ is the well-known golden ratio.

## Consequences of the acTAM for biological systems

As mentioned earlier, $\nu _0$ represents a signal or energetic driving force for ladder elongation, $\tau$ represents randomizing forces or noise in the environment, and the length of the ladder for which the signal is $\ge \tau$ is an artifact produced by the ratio $\nu _0/\tau$, a kind of signal-to-noise ratio for the acTAM. Thus, the ladder is produced by an interaction between its own internal assembly process and its environment. Therefore, it is natural to ask, what is the sensitivity of the structure of the ladder, *i.e.* length, to changes in the environment, i.e $\nu _0/\tau$. For any given $\nu _0/\tau$, a unique ladder length is produced. Nevertheless, there are an infinite number of $\nu _0/\tau$ that can produce a ladder of a given length *n*. Thus, the system itself, the ladders, can only tell us so much about the environment, $\nu _0/\tau$. This is a consequence of the discreteness of the ladder growth in a continuous environment. Using Eq. (19), for a ladder of length $n-1$ to add one additional tile, $\nu _{n-1} = \nu _0/A_{n-1} \ge \tau$, and for growth to terminate at length *n*, $\nu _n = \nu _0/A_{n} < \tau$. Solving both for $\nu _0/\tau$

38 $$\begin{aligned} A_{n-1} \le \frac{\nu _0}{\tau } < A_n \end{aligned}$$

which represents the continuous interval that will produce a ladder of length *n*, spanning a voltage range

39 $$\begin{aligned} A_n - A_{n-1} = \frac{\alpha ^{\prime } (\rho _1^n - \rho _2^n)}{2^n \sqrt{\alpha ^{\prime }(\alpha ^{\prime } + 4)}}, \end{aligned}$$

where $\rho _1$ and $\rho _2$ are given in Eq. (14), the solutions to the characteristic equation. For $\alpha ^{\prime }=1$, Eq. (39) is equal to

40 $$\begin{aligned} \frac{\phi ^{2n}-(1-\phi )^{2n}}{\sqrt{5}}, \end{aligned}$$

which are the even Fibonacci numbers $F_{2n}$. The size of this interval grows unboundedly as *n* becomes large. Thus, larger and larger $\nu _0/\tau$ are required to produce longer ladders, and there is a larger spread in its values between adjacent lengths.

There are two interpretations of this remark. First, the length of the ladder, which is also a measure of the spatial extent over which a signal exceeds the threshold, is the artifact by which the acTAM assembly senses its environment. As the length of the ladder increases, the uncertainty about the $\nu _0/\tau$ that produced the ladder increases, and alternatively, the length of the ladder becomes less sensitive to changes in $\nu _0/\tau$. Autopoiesis^33^ is a theory that describes the ability of a system to maintain itself through a constructive relationship with an environment. In an abstract way, the acTAM captures some of the tradeoffs between a system being able to “know” its environment and also, remaining stable in the face of environmental changes. Living organisms have evolved to find a middle ground of sensing and responding to changes while maintaining those characteristics that define it as a species and have enabled it to survive in a given environment.

## Conclusion

Living systems transduce flows of energy and matter from the environment to grow, produce complex biological patterns, respond to sensory input, and reproduce. Because of their complexity, it is a challenge to model the input-output relationships among the various flows into the system and predict the response, be that a structure or a behavior.

In the original cTAM (Fig. 1), simple circuit tiles attach to a growing ladder circuit if the voltage at the terminus is greater than or equal to a threshold. The cTAM enables self-controlled growth driven by a finite source of energy (voltage source) in a background environment. This complex and characteristic behavior comes as a direct consequence of the interaction among components of the model that are proxies for components in a biological network, namely matter (represented by circuit tiles), energy (input voltages), communication (through signal propagation and dynamic interaction among components representing somatic computation^10,11^ without a central information processor) and interaction with the environment (dynamic growth to homeostasis) at various levels of abstraction.

In this paper, the model has been extended to the acTAM to incorporate other components that are considered important for biological circuit models, such as capacitors and time-varying bioelectric signals, while keeping the model amenable to quantitative and predictive analysis.

Figures 8, 9, 10, 11, 12 and 13 represent results for the acTAM with circuit parameters approximating an axon. After all, the acTAM circuit is highly similar to that of axonal cable theory, which at this stage, is its most appropriate benchmark. In these figures, the acTAM produces a response that approximates signaling in axonal networks, namely it acts as a low-pass filter.

Levin and collaborators^7–9^ describe how complicated distributions of electric potentials might influence biological pattern formation through interactions with genetic mechanisms. The acTAM is a tool that enables exploration of the interaction between electric potentials that depend on the growth of a biological structure, and *vice versa*. Growth itself introduces more complicated time-varying behavior, as seen in Figs. 10, 11 and 12, and additional frequency components in the signal. Interestingly, in Figs. 10 and 12, transients from growth by attachment of circuit tiles results in waveforms that are starting to look like action potentials. It has been suggested^11^ that bioelectric phenomena are an ancient mechanism to control growth and form, possibly predating cells themselves. Transduction of energy to overcome disorder through growth of organized structures would seem to be a prerequisite for early life, and at least in its electric signals, the acTAM starts to demonstrate how those early growth mechanisms may have contributed to the development of complex biological shape and function.

In this paper, the acTAM is monotonic, i.e. once a tile attaches, it cannot detach. Nevertheless, extensions to the acTAM are possible that allow detachment. In order for growth to proceed, the model would have to incorporate forward and reverse reaction rates that would determine the extent of the growth, with this cTAM representing a kind of abstract non-equilibrium thermodynamics that might lend itself to quantitative analysis. Certainly, a series of attachment and detachment events would produce even more complex electric signals and growth behavior.

In conclusion, the acTAM abstracts and is equivalent to other other models of bioelectric networks^7–9^ and offers the potential to uncover deep clues on the etiology of biological structure formation (shape and dynamics). For example, what really controls and limits growth is access to environmental resources and the coupling between components and the environment. It also captures mechanisms that couple growth and electrical signaling in axon-like networks with predictive capability for both length and electrical response. These models also provide full and realistic examples of the well known property of autopoiesis^33^, in which the structure of an organism is determined and preserved by, not in spite of, interaction between components and the environment. Furthermore, the model itself (although not the analysis) is relatively simple and so affords the possibility of models for more complex and realistic phenomena (proteins and neuronal assemblies) in which the representation of matter is less abstract than in conventional models and closer to direct electrical phenomena. These properties would also allow the possibility of providing a more objective and systematic framework to address issues such as homeostasis, self-healing, and other epigenetic biological phenomena as emergent properties^34,35^ rather than being explicitly programmed.

## Data Availability

The datasets used and/or analysed during the current study available from the corresponding author on reasonable request. These include Mathematica programs that were used to generate simulation data for figures.
